# Small intestinal NK/T-cell lymphoma with malignant peritoneal effusion as the first symptom: a case report and literature review

**DOI:** 10.3389/fmed.2025.1610820

**Published:** 2025-07-22

**Authors:** Bing Zhou, Qihan Zhao, Xiaohua Li, Haoru Liu, Hua Hao

**Affiliations:** ^1^Department of Pathology, Second Affiliated Hospital of Jiujiang University, Jiujiang, China; ^2^Jiangxi Provincial Key Laboratory of Cell Precision Therapy, School of Basic Medical Sciences, Jiujiang University, Jiujiang, China; ^3^Department of General Surgery, Second Affiliated Hospital of Jiujiang University, Jiujiang, China; ^4^Department of Pathology, Jiujiang First People’s Hospital, Jiujiang, China; ^5^Department of Pathology, Yangpu Hospital, School of Medicine, Tongji University, Shanghai, China

**Keywords:** small intestinal NK/T-cell lymphoma, malignant peritoneal effusion, T-cell lymphoma diagnosis, treatment, case report

## Abstract

**Introduction:**

Small intestinal NK/T-cell lymphoma (NK/T-L) is a rare condition, and cases presenting with malignant peritoneal effusion as the initial symptoms are not well-documented. We report a unique case that contributes to the understanding of this rare disease.

**Case report:**

A 47-year-old man was admitted to the hospital with a chief complaint of abdominal distension and weight loss persisting for over 2 months. Imaging studies revealed ascites and localized thickening of the small intestine. Paracentesis cytology revealed a significant presence of small-to-medium-sized lymphoid cells. Immunohistochemistry confirmed a diagnosis of T-cell lymphoma. Histological examination confirmed primary NK/T-L of the small intestine after PET-CT excluded metastases from other sites. Despite aggressive chemotherapy, the patient’s condition deteriorated, resulting in his death 4 months later.

**Discussion:**

This case highlights the importance of considering small intestinal NK/T-L in patients with abdominal symptoms and malignant peritoneal effusion. The aggressive nature and poor prognosis of this disease pose challenges in diagnosis and treatment. Increased awareness among clinicians and pathologists is crucial for early detection and improved patient outcomes.

## Introduction

NK/T-cell lymphoma (NK/T-L) is more common, affecting the nasal cavity, with infrequent reports of cases originating in the intestine, whereas NK/T-L presenting with malignant peritoneal effusion is even rarer; to our knowledge, there is only one case that has been reported worldwide (1). Therefore, we report the clinical course, diagnostic challenges, and treatment outcomes of a case of small intestine primary NK/T-L with malignant peritoneal effusion as the first symptom. Through a comprehensive review of the relevant literature, we aim to emphasize the significance of recognizing this atypical presentation and contribute to the body of knowledge for small intestine NK/T-L and raise awareness among clinicians and pathologists, ultimately leading to improved patient outcomes.

## Case report

### Methods

A 47-year-old man presented with a 2-month history of abdominal discomfort and weight loss. The abdominal distension worsened after meals and was occasionally accompanied by non-specific abdominal pain without fever, vomiting, diarrhea, or constipation. The patient underwent abdominal color Doppler ultrasonography, computed tomography (CT), and laboratory examination.

## Results

Abdominal color Doppler ultrasonography showed moderate ascites that did not improve with anti-inflammatory and symptomatic treatments. The patient had no relevant personal or family medical histories and appeared healthy on physical examination. The abdomen was slightly distended with mild tenderness, rebound tenderness, shifting dullness to percussion, and no enlargement of superficial lymph nodes.

**Figure 1:**
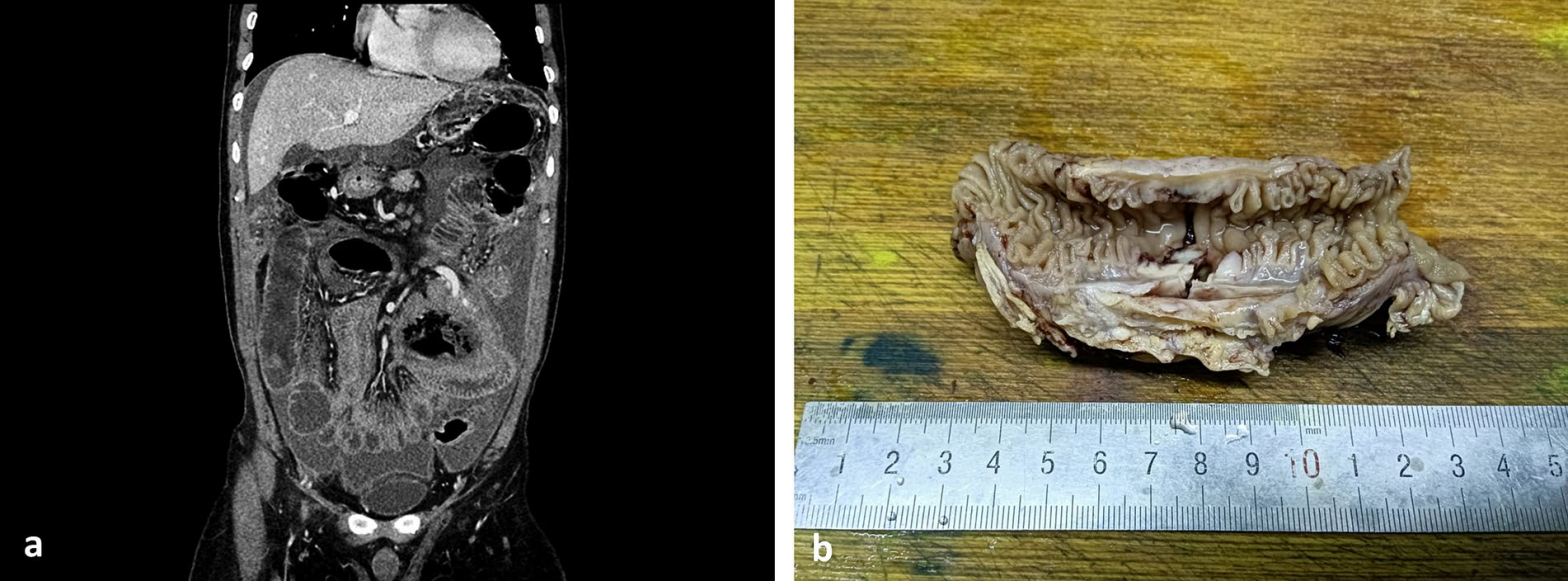
CT image and large body picture of the case. **(a)** CT of the upper abdomen showed peritoneal effusion with small intestinal adhesions, jejunal wall thickening, and multiple abdominal and retroperitoneal lymphadenopathies; and **(b)** ulcer could be seen in the membrane of the small intestine.

Laboratory examination showed elevated levels of C-reactive protein (CRP) at 68.21 mg/L, erythrocyte sedimentation rate (ESR) at 87 mm/h, decreased levels of albumin at 31.7 g/L, and prealbumin at 103 g/L. Quantitative EBV DNA was measured at 2.74 × 10^3^ U/mL. The tumor marker cancer antigen 125 (CA125) was elevated to 104.2 U/mL, and CEA and AFP were normal. The fecal occult blood test yielded positive results. Various tests, including tuberculosis antibody, PPD, ANCA, Vita reaction, hepatitis viruses, blood culture, ENA, and immunoglobulin, were within normal limits. CT of the upper abdomen revealed peritoneal effusion with small intestinal adhesions, jejunal wall thickening, and multiple abdominal and retroperitoneal lymphadenopathies (Figure 1a). Ascites examination showed light yellow, turbid fluid with a positive Rivalta test. Total protein was 36.0 g/L, white blood cell count was 4.021 × 10^9^/L, multinucleated cell ratio was 0.17, lymphocyte ratio was 0.83, ascites Adenosine deaminase (ADA) was 55 U/L, lactate dehydrogenase (LDH) was 730 U/L, and CA125 was 874.6 U/mL. No bacteria were detected by blue staining, and acid-fast bacilli were absent from the smear.

**Figure 2:**
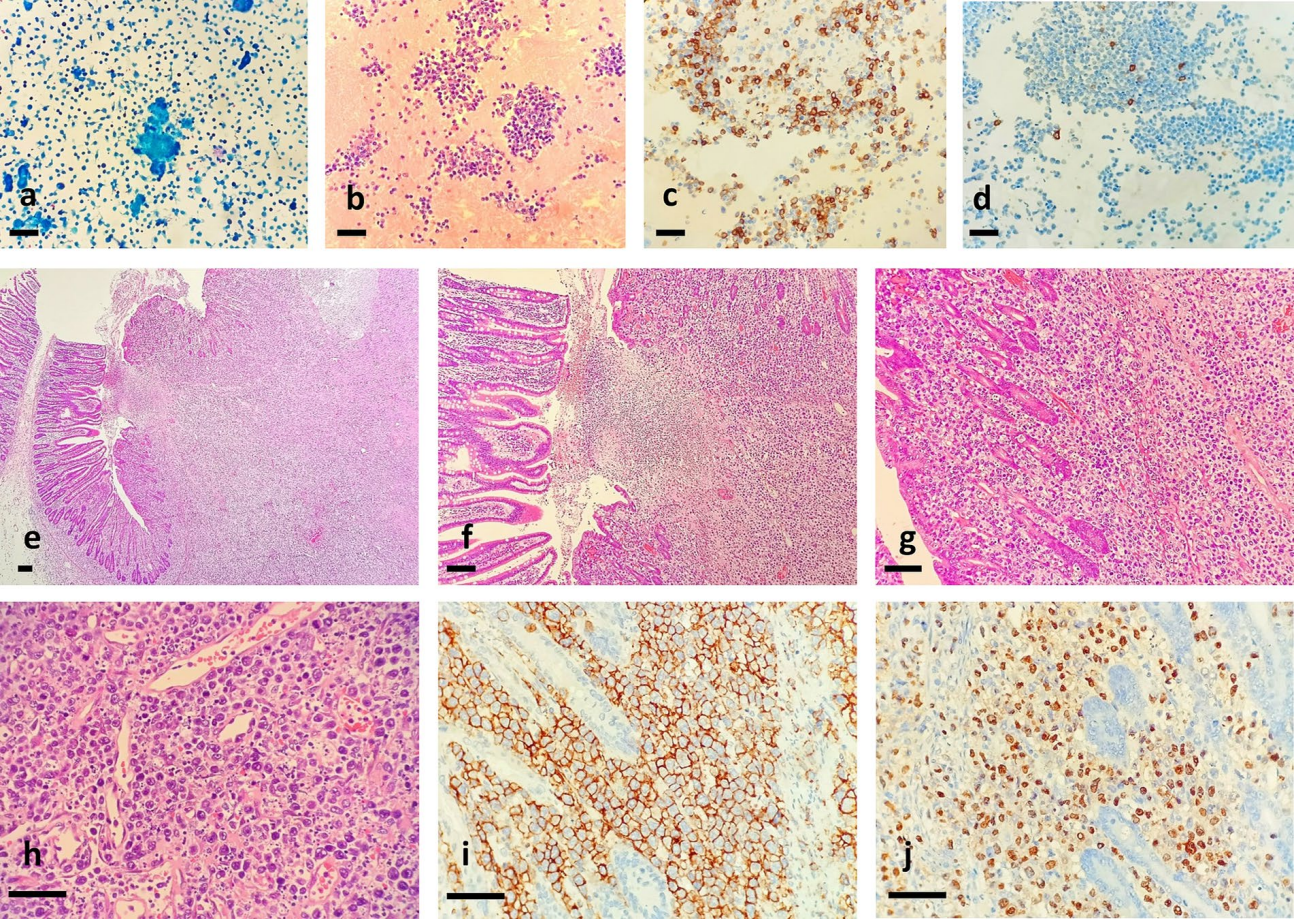
Ascites cytopathological smear revealed diffuse distribution of mildly atypical medium-to-small lymphocytes with scant cytoplasm and deep chromatin, and A section of the bowel revealed full-thickness infiltration of atypical lymphocytes with extensive necrosis in the small intestine. **(a)** Pap staining, **(b)** HE staining for cell mass, **(c)** tumor cells were positive for CD3ε, and D. tumor cells were negative for CD20, bar = 200 μm. (**e**,×40; **f**,×100; **g**,×200; **h**,×400). Immunohistochemical analysis showed that tumor cells were positive for CD56 (**i**,×400). *In situ* hybridization for EBER showed strong positivity in most of the tumor cells (**j**,×400). Bar = 200 μm.

As shown in Figure 1b, ulcer could be seen in the membrane of the small intestine. Ascites cytopathological smear revealed diffuse distribution of mildly atypical medium-to-small lymphocytes with scant cytoplasm, deep chromatin, and cytoplasmic granules that were present in some tumor cells. A large number of necrotic fragments are visible in the background (Figure 2). Microscopic examination of the cell sediment section showed a large number of small lymphocytes with uniform shape, deep chromatin, and irregular and vesicular karyotypes (Figures 2a,b). Immunohistochemical markers were positive for CD3ε(+) (Figure 2c), CD43(+), CD19(−), CD20(−) (Figure 2d), CD5(−), MOC31(−), CK20(−), CR(−), WT-1(−), and Ki67:50%(+), supporting a diagnosis of small bowel T-cell lymphoma. The patient and his family members refused enteroscopy and opted for surgical exploration. Intraoperatively, ulcerative lesions and serosal layer involvement were observed in the jejunum, and the hard part had extended through the serosal layer. A section of the bowel was excised for pathological examination (Figures 2e–h), which revealed full-thickness infiltration of atypical lymphocytes with extensive necrosis in the small intestine (Figures 2e–h). The patient was followed up with CT postoperatively, and PET-CT showed no other lesions. Immunohistochemical staining was positive for CD3ε(+), CD43(+), CD56(+) (Figure 2i), GranB(+), TIA-1(+), and Ki67: 70% (+), whereas it was negative for CD4(−), CD5(−), CD8(−), CD20(−), and EMA(−). *In situ* hybridization detected Epstein–Barr virus (EBV) with positive EBV-encoded small RNA (EBER) staining (Figure 2j), and the molecular testing was positive for TCR-*γ* and negative for TCR-*δ*, leading to the final diagnosis of small intestinal NK/T-L. The patient received two courses of the R-CHOP regimen (cyclophosphamide + doxorubicin + vincristine + prednisone + rituximab) postoperatively but succumbed to disease progression 4 months later.

## Discussion

Extranodal NK/T-L of the nasal type is a rare subtype of non-Hodgkin lymphoma derived from extranodal NK cells and T cells. It accounts for only 0.2–0.4% of all cases and is closely related to EBV infection. Extranodal lymphoma with malignant effusion as the primary feature is rare; the pathological type is mainly B-cell lymphoma (2). Due to the literature on cytological findings related to NK/T-L in effusions is limited, in order to have a comprehensive understanding of the cytomorphology, treatment and outcome characteristics this disease, including our case, we summarized a total of 13 reported cases of NK/T-L with pleural, peritoneal, pericardial, or cerebrospinal effusions that were documented and compared with those reported in the literature (Table 1) (1, 3–11). The male-to-female ratio was 11:3, with a median of 48.5 years. The primary sites were diverse, and the most common nasal cavity with malignant effusion was in only two cases. This may also be related to the fact that NK/T-L in the primary sites, such as the lung, intestine, and pericardium, were closer to the serosal cavity. In 14 cases, 10 cases could not be diagnosed before malignant effusion. This evidence supports that it is difficult to directly diagnose NK/T-L at other primary sites. Our case is similar to the above report, which may be due to the fact that NK/T-L has an insidious onset, a low incidence, and the internal information of deep tumors cannot be effectively obtained.

**Table 1:** Summarization of the clinicopathological features of body cavity effusion involved by NK/T-cell lymphoma.

Author	Age /sex	Site (Primary)	Site (Effusion)	Occur at diagnosis (Effusion)	Cytomorphology	Treatment	Follow-up months	Outcome
Lin et al. (1)	52/M	Small intestine	Pleural+ Peritoneal	Pre-diagnosis	Mononuclear lymphocytes with scanty cytoplasm	Surgery+Chemotherapy	9	DOD
Kumar et al (3)	65/M	Orbital	Pleural	Pre-diagnosis	Mononuclear lymphocytes with folded nuclei, vesicular chromatin and granular cytoplasm	Chemotherapy	NA	DOD
Han et al. (4)	46/M	Adrenal gland	Pericardial	Pre-diagnosis	Medium-sized lymphocytes with clear cytoplasm, irregularly shrunken and hyperchromatic nuclei	Chemotherapy	<1	DOD
Khasawneh et al (5)	48/M	Cardiac	Pericardial+Pleural	Pre-diagnosis	Intermediate to large-sized cells with irregular nuclear contours	Chemotherapy	1	DOD
Walavalkar et al (6)	49/F	Nasal cavity	Cerebrospinal	Post-diagnosis	Medium‑sized lymphocytes with irregular nuclear hyperchromasia and inconspicuous nucleoli	Chemotherapy	3	DOD
Wang et al (7)	40/M	Lung	Pleural	Pre-diagnosis	Medium‑sized lymphocytes with irregular nuclear hyperchromasia	Chemotherapy	<1	DOD
Dunning et al (8)	67/M	Adrenal gland	Cerebrospinal	Pre-diagnosis	Small to large-sized lymphocytes with basophilic cytoplasm, coarse azurophilic granules and prominent nucleoli	Glucocorticoid therapy	<1	DOD
Liu et al (9)	87/M	NA	Pleural	Pre-diagnosis	Medium to large-sized lymphocytes with anaplastic and multilobated nuclei	NA	<1	DOD
Liu et al. (9)	45/F	Nasal cavity	Pleural	Post-diagnosis	Medium to large-sized lymphocytes with anaplastic and multilobated nuclei	Chemotherapy+ Stem cell transplant +Radiotherapy	3	AWD
Liu et al. (9)	77/M	Soft palate	Pleural	Post-diagnosis	Large and anaplastic lymphocytes with multilobated nuclei	Radiotherapy	6	AWD
Liu et al. (9)	44/F	Small intestine	Pleural	Post-diagnosis	Medium to large-sized lymphocytes with anaplastic and multilobated nuclei	Surgery	1.7	DOD
Tai et al. (10)	17/M	Lung	Pleural	Pre-diagnosis	Large‑sized lymphocytes with azurophilic granules	Chemotherapy	2	DOD
Mori et al. (11)	39/M	Lung and liver	Pleural	Pre-diagnosis	Immature lymphocytes with irregular karyotype	Glucocorticoid therapy	1.5	DOD
Our case	47/M	Small intestine	Peritoneal	Pre-diagnosis	Small to medium-sized lymphocytes with deep chromatin and cytoplasmic granules	Surgery+Chemotherapy	4	DOD

M, male; F, female; NA, data not available; DOD, died of disease; AWD, alive with disease.

Intestinal involvement in NK/T-L is even less common, with fewer than 7% of all cases, and clinical manifestations are often non-specific, including abdominal pain, blood in the stool, fever, and weight loss (12). Early endoscopic examination may not yield high positive rates due to submucosal origin, and misdiagnosis as enteritis, intestinal tuberculosis, or inflammatory bowel disease is common in the later stages when polyps or ulcerative lesions develop (13). Ascites with primary features is predominantly caused by abdominal tuberculosis and liver cirrhosis. Different from infection and hypoproteinemia in benign ascites, lymphoma with malignant effusion is mainly caused by advanced lymphoma cells’ direct invasion or metastasis of the serosa and lymph nodes, causing tubal circulation disorders (14). In this case, the patient had no history of tuberculosis or hepatitis, and CT findings showed small bowel wall thickening, adhesions, and multiple abdominal lymph nodes, indicating the possibility of tumor ascites.

Blood routine examination of lymphoma patients often shows mild to moderate anemia. CRP and ESR, as indicators of non-specific inflammatory activity, are both elevated during lymphoma disease activity but lack specificity for diagnosis (15). Ascitic fluid ADA levels ≥40 IU/L showed excellent sensitivity for the diagnosis of tuberculous peritonitis, but a large retrospective study found that lymphoma-related ascites is an important mimic of tuberculous peritonitis that can result in high ascitic fluid ADA levels with similar clinical manifestations (16).

Therefore, when ADA and LDH were increased simultaneously, it was necessary to broaden our thinking and be alert to the possibility of lymphoma. It has been reported in the literature that elevated levels of LDH and CA125 in the serum and ascites are commonly observed in advanced lymphoma cases with intestinal serosal layer involvement, which can reflect the invasion ability of lymphoma (17). The higher the levels, the worse the prognosis, which has important reference value in judging the clinical stage, disease progression, and therapeutic outcome of patients (15). In our case, LDH and CA125 levels were consistent with the reported literature, and quantification of EBV DNA was significantly increased, indicating a high suspicion of NK/T-L with ascites.

Cytological examination of ascitic fluid is a mature diagnostic technology, with malignant cells observed in 60% of malignant ascites. A large sample study reported that in 197 samples of patients with lymphomatous effusion, the positive rate of malignant cells found in cytology was 56.6%. It is confirmed that there are some missed diagnoses and misdiagnoses, which are related to the collection of tumor cells and the diagnosis level of pathologists (18). The cytologic smears of the Extranodal NK/T-cell lymphoma (ENKTL) effusion specimens were highly cellular, and the tumor cells were small- to large-sized with pleomorphic nuclei and coarse chromatin. A moderate amount of eccentric basophilic cytoplasm and azurophilic granules was also seen (Table 1) (1, 3–11). Microscopic cytological examination of the ascitic fluid in this case showed many small and medium lymphocytes with consistent morphology and some atypia. Proliferative lymphocytic lesions were also considered. The patient had no history of lymphoma, and it was difficult to diagnose lymphoma by cytology alone. After the cell sediment was collected by centrifugation, T-cell lymphoma was diagnosed based on cell morphology and immunohistochemical markers. Therefore, ascites of unknown cause should be checked with a cell wax block as much as possible, which can not only determine benignity and malignancy but also be combined with immunohistochemical markers to identify common adenocarcinoma cells, mesothelial cells, and lymphocytes in ascites. The final diagnosis of ENKTCL requires the help of histology, which shows diffuse and consistent lymphocyte infiltrating growth in the small intestinal wall, and vascular destruction and necrosis as its unique histological features; immunohistochemical markers CD2, CD3ε, CD56, TiA-1, granzyme B, and perforin positive, and EBER *in situ* hybridization positive can be definitively confirmed (19). The differential diagnosis of small intestinal NK/T-L is particularly important because of the differences in treatment and prognosis. It is necessary to differentiate from intestinal inert T-lymphoproliferative disorders, Enteropathy-associated T-cell lymphoma (EATL), Monomorphic–epitheliotropic intestinal T-cell lymphoma (MEITL), peripheral T-cell lymphoma of the gastrointestinal tract, angioimmunoblastic T-cell lymphoma, and B-cell lymphoma. These are lymphoid disorders occurring in the small intestine and are associated with clinical histories, defined microscopic morphology, specific immunohistochemical markers, EBERs, and TCR gene detection, which can help us make a final diagnosis.

**Figure 3:**
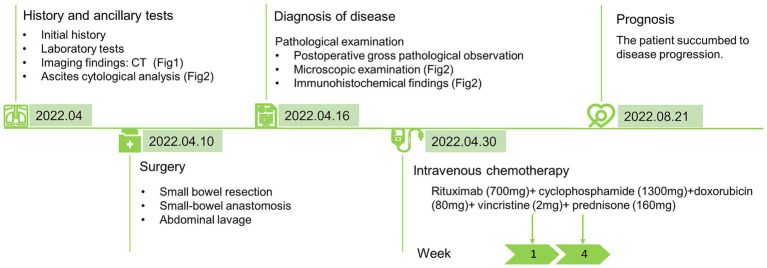
Timeline figure summarizes the case diagnosis and treatment pathway.

Currently, there are no standard treatments for NK/T-L. The total effective rate of the conventional Cyclophosphamide, Hydroxydoxorubicin, Oncovin and Prednisone (CHOP) regimen is approximately 36%, and the total effective rate for relapsed and refractory patients is less than 10% (20). In this case, the patient received two courses of the R-CHOP regimen but succumbed to disease progression rapidly 4 months later, which might be related to the failure to select the standardized treatment. CHOP chemotherapy is not an optimal treatment for NK/T-L, and L-asparaginase-containing chemotherapy is a key component of first-line treatments for systemic NK/T-L. Standard treatments for refractory or relapsed NK/T-L, dexamethasone, methotrexate, ifosfamide, l-asparaginase, and etoposide (SMILE) regimen and sequential chemoradiotherapy is recommended (21), or hematopoietic stem cell transplantation may be considered (22). The NK/T-L with malignant effusion was in the advanced stage of the disease. In the 14 patients we collected, it was found that the treatment effect was not significant, whether it was surgery, chemoradiotherapy, or stem cell transplantation. Recently, next-generation sequencing (NGS) was performed in ENKTL, and the missense mutation of STAT3, STAT5B, and RNA helicase gene *DDX3X* was found, which may be potential therapeutic targets for treatment (7, 23). Given that NK/T-L can destroy intestinal adhesions, when patients present with unexplained abdominal pain and hematochezia, conventional diagnostic methods such as traditional colonoscopy fail to identify the cause. Consideration should be given to performing small bowel endoscopy, and biopsy can be considered to effectively improve the early detection rate of lesions. Nevertheless, the prognosis of small-bowel NK/T-L remains poor, with a median survival time of only 3–6 months (24, 25). The patient received R-CHOP chemotherapy after surgery and died of secondary symptoms 4 months later. A timeline figure summarizing the case diagnosis and treatment pathway is shown in Figure 3.

In summary, the clinical manifestations of small-intestinal NK/T-L are atypical, with ascites being the first symptom. When refractory ascites is accompanied by elevated serum LDH, CA125, and EBV, lymphomatous disease should be considered after excluding other common diseases. The possibility of ascites, especially NK/T-L, combined with pleural and ascites cytology to provide diagnostic clues, active deep colonoscopy biopsy or laparoscopy, and finally combined with immunohistochemical staining and EBER *in situ* hybridization should be considered to establish a pathological diagnosis and provide clinical treatment.

## Data Availability

The original contributions presented in the study are included in the article/supplementary material, further inquiries can be directed to the corresponding author/s.
